# Solvent front position extraction with semi-automatic device as a powerful sample preparation procedure to quantitatitation of tryptophan in human plasma

**DOI:** 10.1038/s41598-020-71846-6

**Published:** 2020-09-15

**Authors:** Anna Klimek-Turek, Adam Chomicki, Emilia Fornal, Anna Pradiuch, Michał Hys, Tadeusz H. Dzido

**Affiliations:** 1 Physical Chemistry Department, Medical University of Lublin, Chodźki 4A, 20-093 Lublin, Poland; 2 Department of Pathophysiology, Medical University of Lublin, ul. Jaczewskiego 8b, 20‐090 Lublin, Poland; 3 I Chair of Anaesthesiology and Intensive Therapy with Clinical Paediatric Department, Medical University of Lublin, Jaczewskiego 8, 20-954 Lublin, Poland

**Keywords:** Medical and clinical diagnostics, Analytical chemistry, Mass spectrometry

## Abstract

In the paper the results of the tryptophan determination in human plasma samples prepared with the novel Solvent Front Position Extraction (SFPE) technique are presented. The SFPE procedure is used for preparation of real biological sample for the first time. The results obtained using SFPE are compared with those using the classical sample preparation procedure. Under the optimal conditions, tryptophan and its internal standard were separated from other plasma compounds (matrix) as a small common zone/spot on a chromatographic plate using semiautomatic device equipped with moving pipet, which distributed developing solvent on the adsorbent layer. Tryptophan and the internal standard were evenly distributed within the small common zone from that the both substances were extracted and the solution obtained was transferred to quantitation with LC–MS and MS techniques. The determination results are satisfactory, the percentage values of relative error and RSD relative standard deviation do not exceed 5%. The procedure is characterized by simplicity, high analysis throughput, very good sample purification and seems to be easy applicable to other biological samples with these advantages mentioned.

## Introduction

The qualitative and quantitative analysis of substances in body fluids plays a significant role in diagnostics^1–3^. A presence, absence, or change in concentration of some components could be used as an indicator of a pathological process in an organism^4,5^ and/or gives information about effectiveness of a particular therapy/treatment^2,6–8^. However, analysis of body fluids constituents present a number of challenges, especially during the sample preparation stage^9,10^. From the point of view of chemical analysis, biofluids consist of proteins, peptides and a variety of substances and their metabolites. For this reason the preparation stage is inevitable prior to the quantitation of a given component/s.

The properly carried out the sample preparation stage allows avoiding problems associated with contamination of the measuring device but also guarantees reproducible and reliable results^11^. In case of plasma samples, protein precipitation is the most commonly used sample preparation method because of its low cost and minimal equipment requirements. The plasma proteins are frequently removed through pretreatment of sample with acids: perchloric acid^12–14^, trichloroacetic acid^15–17^, hydrochloric acid^18^ or alcohols: methanol^13,19^ and ethanol^20^. Unfortunately, this method has relatively poor sample cleanup, what is very unfavorable, especially in the case of LC–MS/MS analysis, where co-eluting components can interfere with ionization process of target substances (matrix effect)^11^. One should be aware, that although there are many methods for determining substances in body fluids^19,21–28^ the High Performance Liquid Chromatography coupled with Mass Spectrometry (LC–MS/MS) has become the most popular^29^. That is why it seems so important to find a method of sample preparation that will purify them to a large extent, without extending the time of analysis or making the analysis tedious.

In our previous papers we proposed the modern approach to sample preparation procedure, especially for biological samples^30–32^. The procedure is named as the Solvent Front Position Extraction (SFPE)^31^ and is based on the following steps^30–32^: (a) the internal standard (IS) is added to the sample, (b) a drop of the sample solution is applied on the chromatographic plate, (c) the chromatogram is developed to locate the substance/s of interest and internal standard/s zones at the final solvent front position, (d) the substance/s and internal standard are extracted from this zone (or its part) and transferred to the instrumental device for quantitation. The procedure was tested, with very satisfactory results, for preparation of samples of serum matrix, which was fortified with the solute of interest (acetaminophen) and internal standard (acetanilide)^30^. Next, the analogous procedure was tested to a more complex sample containing 8 substances characterized by different properties^31^. By finding the appropriate chromatographic system during screening tests and overcoming the problem of the radial chromatography at the stage of application of the sample, all substances of interest were successfully determined with an error of less than 5%^33^. Additionally, the procedure has been streamlined by partially automating it by the application a prototype device equipped with moving pipette delivering a solvent to the adsorbent layer^34,35^. In our previous research we have proven that our sample preparation procedure is accurate, repeatable and (when the prototype automation is applied) also very user-friendly^33,35^. However, so far we have not tested the procedure on “real samples”, taken directly from patients. This is a necessary step if one wants to propose a method that is intended for the preparation of biological samples^36^. Therefore, we decided to use the SFPE procedure to prepare plasma samples from patients for the quantification of tryptophan. This exogenous amino acid is constantly attracting the attention of scientists due to the significant role it plays in the human body^37^. In addition the principal role of tryptophan as a constituent of protein synthesis, it is also the precursor of numerous physiologically crucial substances such as neuroactive serotonin, melatonin, tryptamine, or immunosuppressive kynurenine^12,38–42^. Due to its involvement in so many metabolic pathways, tryptophan influences on mood, sleep and is associated to stress response and appetite regulation^38,43,44^. The interest of scientists is also attracted by a possible role of kynurenine pathway in cancer treatment, because this substance is able to suppress anti‐tumor immune response^45^. Measurements of tryptophan concentration in biological samples are not easy because of its lability and relatively low physiological level. The presence of many interfering substances in samples also negatively affect the tryptophan determination (matrix effect)^10^. As with most plasma-assayed substances, the most frequently chosen sample preparation method for quantitation of tryptophan is protein precipitation. Unfortunately, as it was mentioned above, this procedure does not purify the serum samples from contaminants at a level appropriate for LC–MS analysis.

In our paper we want to present the results of the tryptophan determination in biological samples prepared with the novel Solvent Front Position Extraction technique. Moreover, these results are compared with those, obtained using the classical sample preparation procedure. This is the first time that the SFPE procedure has been used to prepare patients samples. The obtained results show that the procedure used can be successfully applied to the preparation of plasma samples for the quantitative determination of tryptophan, and in a perspective, for the preparation of samples for the determination of many other substances.

## Results and discussion

### Classical procedure of sample preparation

If a new technique is to be launched, it is necessary to compare it with a well-grounded one in the laboratory practice. Then it is necessary to know how much the novel technique differs from the old one if we compare the results in respect of quantification, but also in the aspects of the time and cost incurred. One of the most commonly used sample preparation approaches for biofluids is protein precipitation^12–20^. The popularity of this method comes from its simplicity and relatively low cost (compared for example to the SPE). From this reason, in our research we decided to choose this sample preparation method for LCMS analysis as a “classical procedure”. The results of tryptophan quantitation in samples obtained in this way, in the further part of our research are treated as “true'' or ''reference values”.

**Figure 1:**
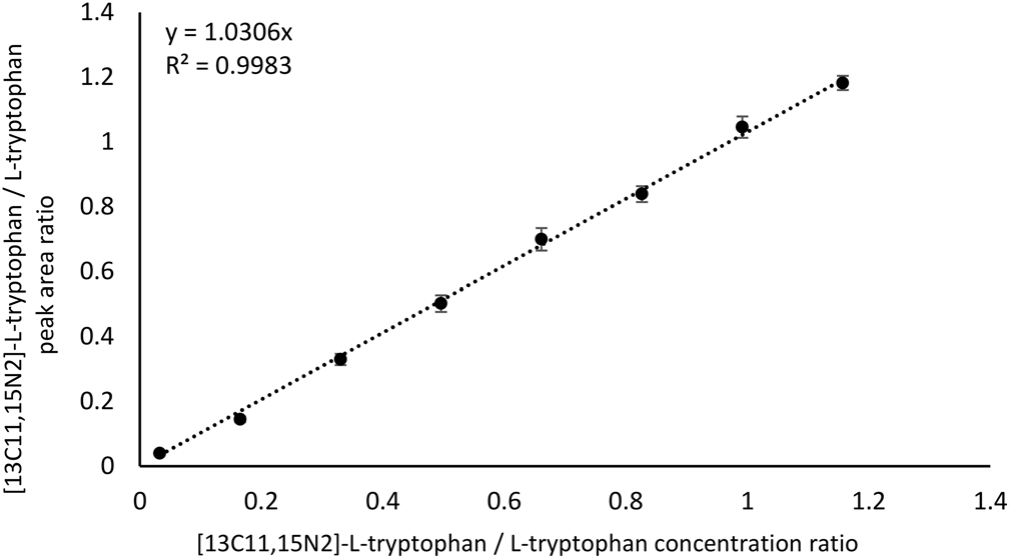
Calibration curve for peak area ratio of [13C11,15N2]-L-tryptophan / L-tryptophan versus [13C11,15N2]-L-tryptophan / L-tryptophan concentration ratio in human plasma. Classical procedure.

Under conditions described in Methods part, tryptophan was determined by measuring the internal standard/analyte peak area ratio. A plot of mean internal standard/analyte/peak area ratio against the concentration ratio of labeled tryptophan and tryptophan was constructed (Fig. 1). The linear relationship (R^2^ = 0.9983) over the concentration range from 0.5 to 17.5 µg/mL was obtained. The limit of quantitation (LOQ) and the limit of detection (LOD) were calculated using the formula: LOD = 3.3σ/s and LOQ = 10σ/s, respectively, where σ is the standard deviation of the response, s is the regression line slope. The values of limit of detection and limit of quantification were 0.50 µg/mL and 1.51 µg/mL, respectively. The tryptophan concentrations in the samples determined based on these calibration curve were as follows: 10.46 µg/mL, 6.91 µg/mL and 11.40 µg/mL (Table 1).

**Table 1:** The figures of merits of the standard procedure.

	Sample 1	Sample 2	Sample 3
Tryptophan concentration (µg/mL)	10.46	6.91	11.30
% RSD	0.86	0.11	0.69
LOD (µg/mL)	0.50		
LOQ (µg/mL)	1.51		

### SFPE procedure of sample preparation

#### SFPE procedure optimization

As it was presented in our previous papers^30–32,35^ the precision and accuracy of the SFPE technique are based on the premise that both sample components and the internal standard after chromatogram developments were evenly distributed in the focused common zone of the final solvent front position. The obtaining of the final solvent front position was preceded by additional chromatogram development which allowed cleaning the sample solution from matrix components showing weaker and stronger retention than the solutes of interest. To obtain this effect, a suitable eluent is necessary to develop a chromatogram of sample mixture that gives the substance and the internal standard retardation factor values close to 0.5. Therefore, before main experiments, it is essential to formulate relationships between the retention of analytes and the chromatographic system composition. Afterward for this reason, different TLC adsorbents (Silica gel, C18, CN, NH2, Diol) were tested using a wide range of solvents (toluene, acetonitrile, water, methanol, ethyl acetate) and their mixtures. The influence of pH on retention was also examined. As a results of the screening study, the Diol chromatographic plate was identified as the best stationary phase. The retardation factor, R_f_, values around 0.5 were achieved with mobile phase consisted of 35% toluene in methanol with the 0.1% addition of formic acid. In turn, the R_f_ coefficient equal to 1.0 for the substances is achieved in the chromatographic system with the mobile phase comprised methanol with addition 0.1% formic acid.

**Figure 2:**
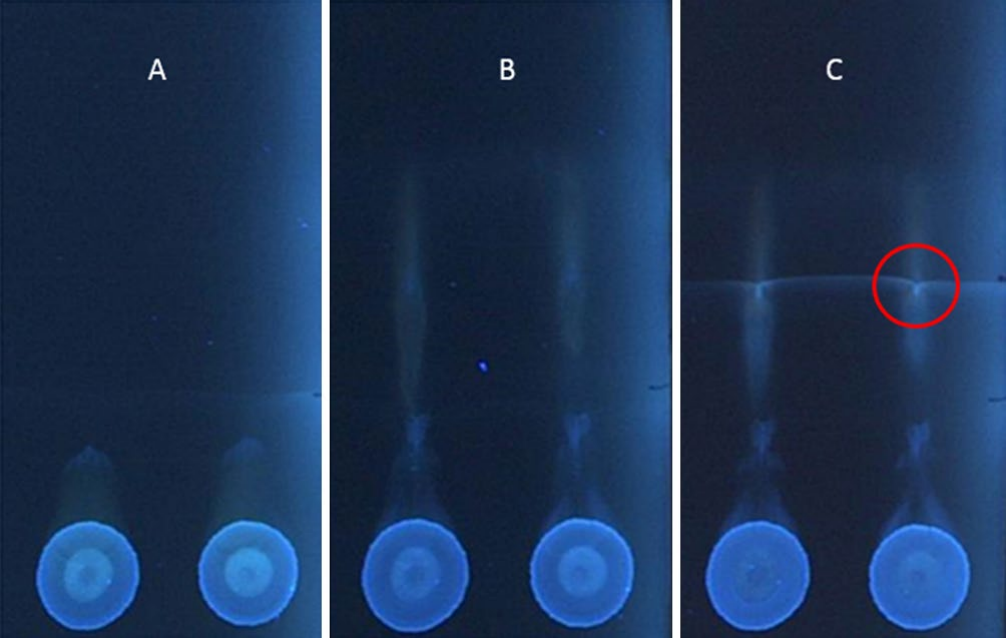
The plasma sample chromatograms. (**A**) after development performed by methanol with 0.1% formic acid, (**B**) after development performed by 35% toluene in methanol (with 0.1% formic acid), (**C**) after development performed by methanol with 0.1% formic acid. Stationary phase: HPTLC Diol F254. Λ = 366 nm. The position of the tryptophan and its internal standard is marked with a circle. See discussion in the text.

The stages of SFPE sample preparation procedure for tryptophan quantitation in a human plasma are presented in Fig. 2. Our previous research^33^ showed that in order to elute substances from the serum/plasma/blood zone, a mobile phase with high concentration of an organic modifier should be used, so the first development is performed by methanol with 0.1% formic acid. This development aims at the elution of the substance from the plasma zone, so it can be carried out over a short distance (Fig. 2A). Next, the chromatogram is developed using a 35% toluene in methanol (0.1% formic acid) solution which provides the R_f_ value for the substance and the internal standard about 0.5. As a result, the target substances are separated from the matrix components, which possess higher and lower adsorption energy than the substances of interest (Fig. 2B). During the last stage the chromatogram is developed using methanol with addition 0.1% formic acid in order to make the tryptophan and its internal standard zones located at the common solvent front final position (Fig. 2C). From this area target substances are extracted to vials or directly to a mass spectrometer. The chromatogram presented in Fig. 2C shows that using the SFPE sample preparation procedure, the target substances are very well separated from the matrix components.

### Variants of analysis with SFPE

#### SFPE-vials-LCMS

**Figure 3:**
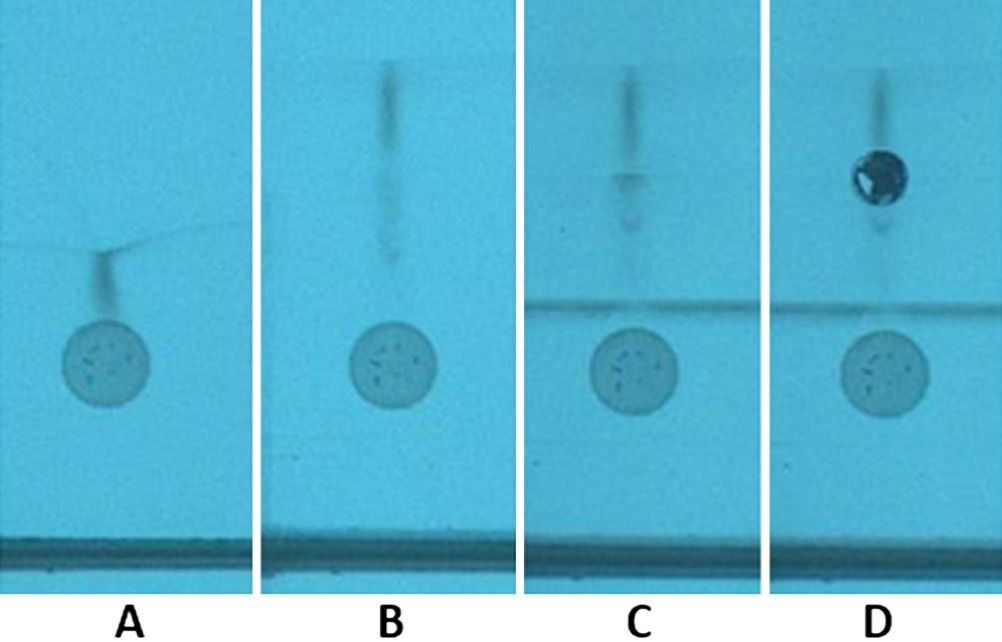
The plasma sample chromatogram. (**A**) after development performed by methanol with 0.1% formic acid, (**B**) after development performed by 35% toluene in methanol (with 0.1% formic acid), (**C**) after development performed by methanol with 0.1% formic acid, (**D**) after tryptophan and its internal standard extraction by water with 0.1% formic acid using TLC-MS Interface. Stationary phase: HPTLC Diol F254. Λ = 254 nm.

**Figure 4:**
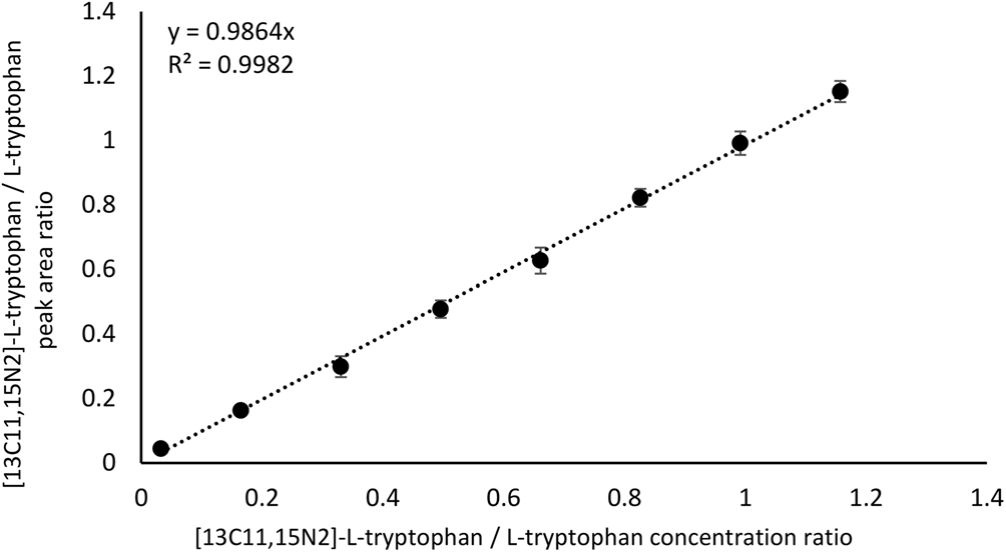
Calibration curve for peak area ratio of [13C11,15N2]-L-tryptophan / L-tryptophan versus [13C11,15N2]-L-tryptophan / L-tryptophan concentration ratio in human plasma. SFPE-vials-LCMS procedure.

The SFPE-vials-LCMS variant was as follows. First, the samples of plasma were applied on the chromatographic plate. The chromatograms were developed using a new prototype device according to the procedure described in “Experimental” section. The examples of chromatogram are presented in Fig. 3. After chromatograms developments the substances located in a small zone of the final front position were extracted into the vials. The vials were placed into the autosampler, then the LCMS analysis was performed. The obtained calibration curve is shown in Fig. 4. One can see that the response is linear across the whole measurement range. The values of R^2^ = 0.9982 are alike that in case of the reference procedure. The results of tryptophan quantitation are gathered in Table 2. Comparing the results to those obtained for samples prepared by the classical procedure one can see a high consistency of the results. The relative error defined as 100(M_SVLM_ − sLCMS)/sLCMS where sLCMS is the reference value (value of tryptophan concentration (µg/mL) determined in samples after protein precipitation procedure combined with LCMS analysis) and M_SVLM_ is the value of tryptophan concentration determined in samples after SFPE procedure combined with LCMS analysis, does not exceed 5%. The relative standard deviation value is also low and does not exceed 2.3% (Table 2).

**Table 2:** The figures of merits of the SFPE-vials-LCMS procedure.

	Sample 1	Sample 2	Sample 3
% Relative error	− 4.60	− 2.28	0.60
% RSD	1.18	2.26	1.50
LOD (µg/mL)	0.53		
LOQ (µg/mL)	1.61		

Since the results of the tryptophan determination with two discussed procedures are comparable, it can be concluded that the SFPE technique is as good for preparing samples for quantitative analysis as the reference method.

**Figure 5:**
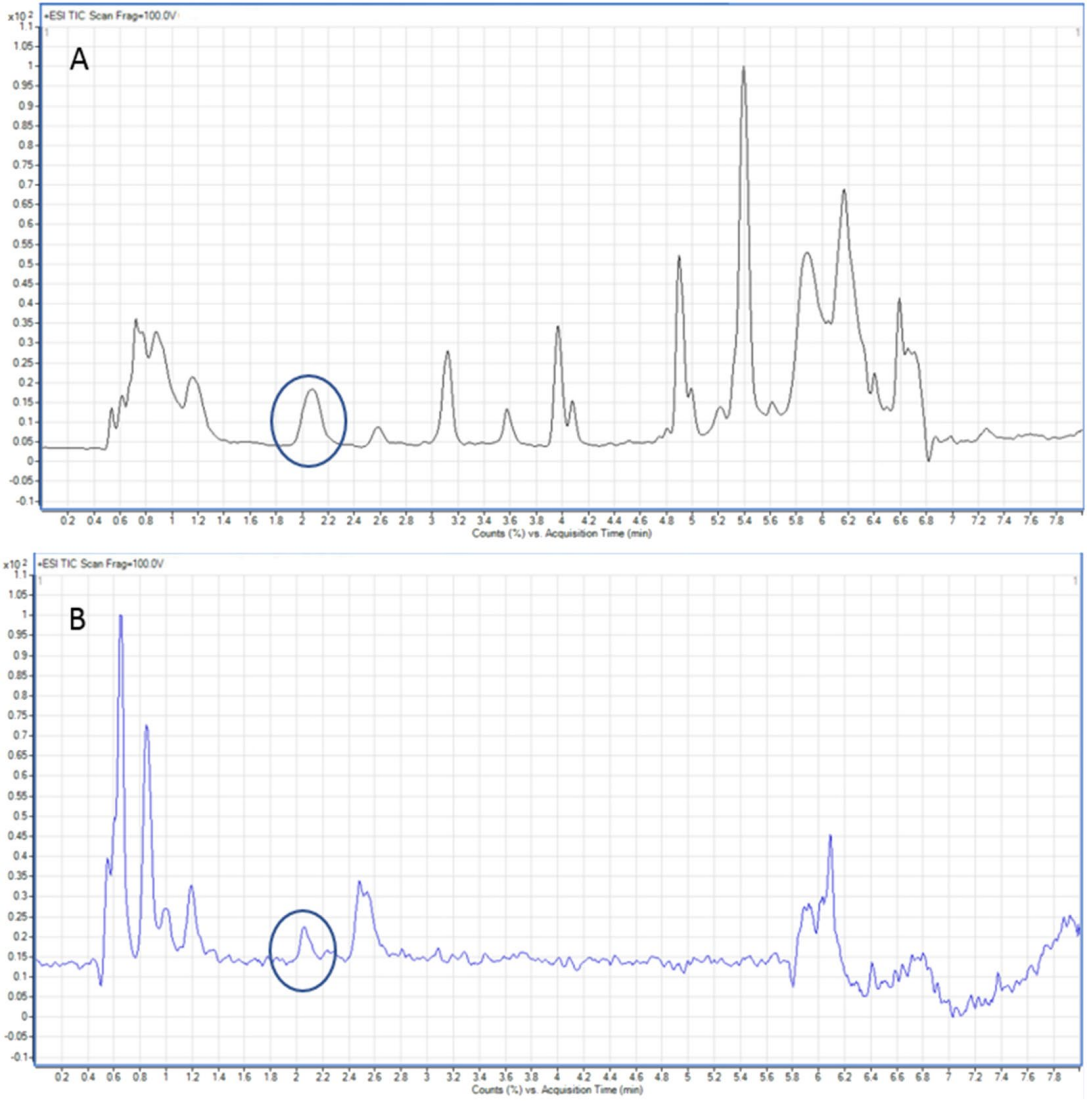
LC–MS scan mode chromatogram of human plasma sample prepared using (**A**) the reference sample preparation procedure, (**B**) SFPE sample preparation procedure. The peaks of the tryptophan and its internal standard are marked with a circle.

The use of the SFPE technique instead of the protein precipitation procedure before LCMS analysis does not significantly reduce the time or costs of the analysis (a more detailed discussion of this issue is at the end of the section). However, the sample prepared by the SFPE technique is much more purified in respect of interfering substances compared to the samples prepared by the classical technique. This is confirmed in Fig. 5 which presents the results of LC–MS analysis in the scan mode performed for samples prepared using the protein precipitation (5A) and SFPE (5B) procedures. To remove contaminants peaks (from solvents, instruments ect.) the blank signal was subtracted from the observed analytical signal. One can notice that the classical sample preparation procedure gives numerous peaks corresponding to impurities. The sample prepared using the SFPE technique is free of most matrix components. At this point it is worth recalling that the chromatographic system was the same for both analyzed samples, so any differences visible on the chromatograms are only related to the difference in the qualitative and quantitative composition of the samples resulting from different methods of their preparation for instrumental analysis. The difference in the chromatograms is especially visible when the concentration of the organic modifier in the mobile phase increases (from 5 to 8 min). In the case of the sample purified by the classical procedure, many impurities are visible on the chromatogram, which in the case of the sample purified by the SFPE procedure are absent.

Lower signal obtained for tryptophan in the sample prepared by the SFPE technique is due to the fact that it was prepared with more than 10 times less volume of plasma in comparison to that prepared with the classical procedure.

#### SFPE-vials-MS

The comparison of the results of the substance quantitation in the samples prepared with the protein precipitation and SFPE techniques shows that the results of the determination are very similar. This means that SFPE technique can be successfully used to prepare biological samples for the determination of tryptophan. During the LC–MS analysis, the most time is devoted to the chromatographic (HPLC) separation stage. However, this step is very desired, especially in the case of biological samples with a rich matrix. Unwanted compounds coeluting with target substances may adversely affect on quantitation results (so-called matrix effect). This effect is especially undesirable if substances of interest are at low concentrations.

As was mentioned above, the use of SFPE sample preparation significantly reduces the amount of matrix contaminants and, thereby, the matrix effect is minimized. For this, it is possible to skip the chromatography (HPLC) stage before MS determination without the loss of accuracy and precision of the analysis.

**Figure 6:**
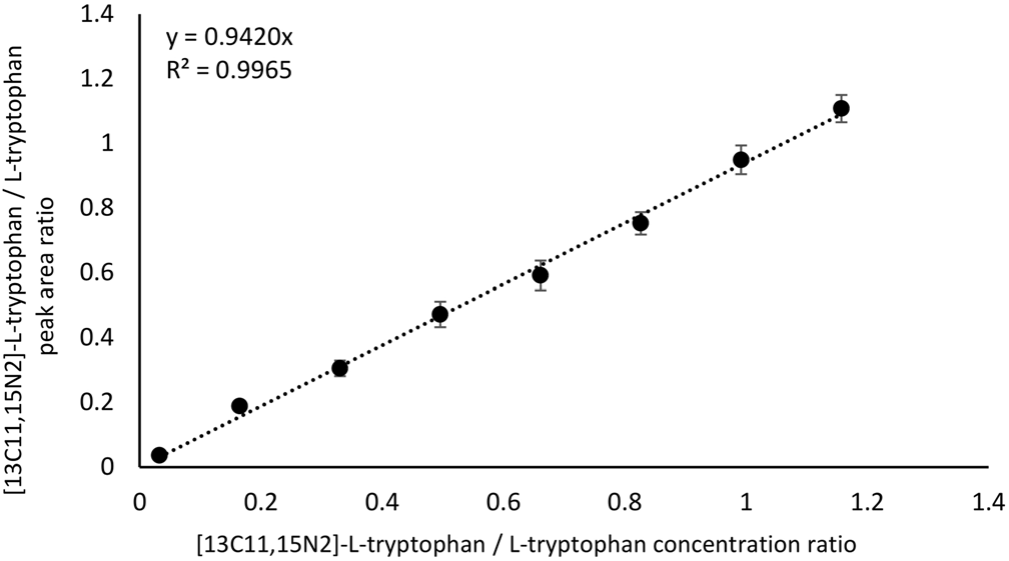
Calibration curve for peak area ratio of [13C11,15N2]-L-tryptophan / L-tryptophan versus [13C11,15N2]-L-tryptophan / L-tryptophan concentration ratio in human plasma. SPFE-vials-MS procedure.

At this stage of the research, after the final chromatogram development in SFPE procedure, the substances were extracted from their final position of the mobile phase front to vials and then introduced into MS instrument. The obtained calibration curve is presented in Fig. 6. It can be seen that the response is linear from 0.5 to 17.5 µg/mL and the R factor close to 1.0 (R^2^ = 0.9965), as in case of calibration curves discussed earlier. The determined tryptophan concentration values in the analyzed samples are close to the values obtained using the classical sample preparation technique. The relative error, defined as 100*(M_SVM_ − sLCMS)/sLCMS where M_SVM_ is the value of tryptophan concentration determined in samples after SFPE procedure combined with substances extraction to vials and MS analysis, does not exceed 5%, neither does the RSD value (Table 3). The LOD and LOQ values are slightly higher compared to the previously discussed procedures.

**Table 3:** The figures of merits of the SFPE-vials-MS procedure.

	Sample 1	Sample 2	Sample 3
% Relative error	− 3.89	4.03	− 0.40
% RSD	3.52	3.70	4.37
LOD (µg/mL)	0.77		
LOQ (µg/mL)	2.34		

In the case of LC–MS, the signal corresponding to tryptophan is registered for ~ 30 s. When the column chromatography stage is omitted, maintaining the same eluent flow rate, the residence time is 10 s, so the ions are counted for a shorter period of time. Optimization of the mobile phase flow rate should improve the sensitivity of the analysis.

#### SFPE-MS

**Figure 7:**
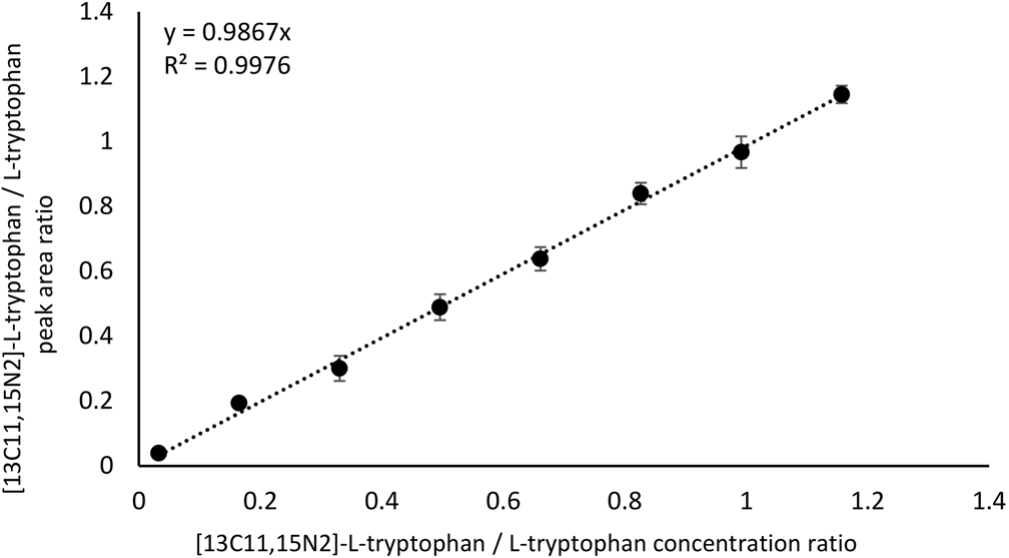
Calibration curve for peak area ratio of [13C11,15N2]-L-tryptophan / L-tryptophan versus [13C11,15N2]-L-tryptophan / L-tryptophan concentration ratio in human plasma. SFPE-MS procedure.

The preparation of samples using the SFPE procedure, which is based on liquid–solid phase extraction of analytes from adsorbent layer, gives the opportunity to introduce the substances directly from the plate to the mass spectrometer using the TLC-MS Interface. This procedure is worthy of investigation due to expected considerable shortening the time of analysis and its cost in comparison to the procedure with HPLC involved. The calibration curve prepared using this approach shows linearity in the analyzed concentration range (R^2^ = 0.9976, Fig. 7).

The % relative error defined as 100*(M_SM_ − sLCMS)/sLCMS (where M_SM_ is the value of tryptophan concentration determined in the samples after SFPE procedure combined with MS), and %RSD do not exceed 5%. LOD and LOQ are comparable to those obtained by the variant SFPE-vials-MS and are 0.64 µg/mL and 1.95 µg/mL, respectively (Table 4).

**Table 4:** The figures of merits of the SFPE-MS procedure.

	Sample 1	Sample 2	Sample 3
% Relative error	4.80	0.70	2.52
% RSD	4.47	2.06	4.80
LOD (µg/mL)	0.64		
LOQ (µg/mL)	1.95		

### Comparison of procedures

The comparison of all described procedures/approaches regarding analysis time and solvent consumption is presented in Table 5. The calculations were carried out for the protein precipitation procedure involving 24 samples (24 samples could be centrifuged simultaneously) and 32 samples in the case of the SFPE procedure (limitation due to the size of the chromatographic plate). One can notice a significant reduction of the analysis time for the SFPE-vials-MS and the SFPE-MS procedures in comparison to the classical/reference and SFPE-vials-LCMS ones. It should be emphasized here that in the case of SFPE-vials-LCMS procedure the analysis time could be shorter than 16.5 min. It should be remembered that the sample after SFPE is very well purified, so the gradient used to get rid of interfering substances from the column is no longer necessary, and, thus, the column equilibration stage can also be omitted. In this case, the analysis time would be 7.5 min.

**Table 5:** Time of analysis and solvent consumption using different procedures of sample preparation applied in the research.

	Standard/reference procedure	SFPE-vials-LCMS	SFPE-vials-MS	SFPE-MS
**Sample preparation step**				
Time per sample (min)	2.5	3.0	3.0	2.3
Automation	No	Partial		
**Quantitative analysis**				
Time per sample (min)	13.5 (flushing and injection + analysis + column flushing by gradient + column equalibration)	13.5 (*4.5) (flushing and injection + analysis + column flushing by gradient + column equalibration)	2.0 (flushing and injection + analysis)	2.0 (analysis + flushing)
Total analysis time (min)	16.0	16.5 (*7.5)	5.0	4.3
Average consumption of solvents (mL)	150	150 (70)	60	35

The use of the SFPE-vials-MS procedure instead of classical one for tryptophan quantitation reduces the time of analysis from 16.0 to 5.0 min. Also the cost of analysis with the SFPE-vials-MS technique is lower compared to the classical/reference procedure, due to almost three time lower solvent consumption and no need to buy a chromatographic column. However, a procedure that deserves special attention is the SFPE-MS one. The analysis time in this case is very short, it is about 4 min per sample, almost 4 times shorter in comparison to the classical procedure. Solvent consumption is also very low, approximately 4 times lower than in the classical procedure. Last but not least, the tryptophan determination procedure carried out in this way is very easy to be fully automated.

## Experimental

### Materials and reagents

Chromatographic plates, HPTLC Diol F254, 10 × 20 cm, were supplied by Merck (Darmstadt, Germany). Methanol and toluene LC–MS grade were purchased from Merck (Darmstadt, Germany). Acetonitrile LC–MS grade was purchased from Fisher Chemical (Waltham, MA, USA). Formic acid (LC–MS grade) was purchased from Merck (Darmstadt, Germany). The ultra-pure water was obtained from Millipore Direct-Q3- UV purification system (Merck, Darmstadt, Germany). Tryptophan and [13C11, 15N2]-L-tryptophan were purchased from Alsachim (Illkirch Graffenstaden, France). Blood samples were collected from volunteers.

### Plasma sample preparation

#### Common stage of experiments

Blood samples collected from volunteers were subjected to centrifugation. The obtained plasma samples were stored at -80 °C until analysis. Next the frozen plasma samples were thawed at temperature 4 °C. Then, the internal standard ([13C11, 15N2]-L-tryptophan) was added to the plasma samples so that its final concentration was 12.5 µg/mL. The samples prepared in this way were divided into two parts, each of them was subjected to quantitative analysis according to two different protocols: classical procedure and SFPE procedure.

##### Classical procedure

*Calibration curve*.

The stock solution of stable isotope labelled L-tryptophan standard was prepared in acetonitrile: methanol (1:1 v/v) at the concentration of 1 mg/mL. The working solution of stable isotope labelled L-tryptophan was prepared by tenfold dilution of the stock solution with water. Samples for calibration were prepared by the addition of appropriate aliquots of the working solutions to 200 µl of plasma containing known, previously determined, tryptophan concentration. The final concentrations of stable isotope labelled L-tryptophan are presented in Table 6. Calibration curves were prepared by plotted ratio of the IS signal to the analyte versus the IS/analyte concentration.

**Table. 6.** Calibration curve.

Calibration level	[13C11, 15N2]-L-tryptophan concentration (µg/mL)
1	0.5
2	2.5
3	5
4	7.5
5	10
6	12.5
7	15
8	17.5

Protein precipitation and metabolite extraction were performed by adding 1 volume of plasma (0.2–0.5 mL) to 3 volumes of cold (− 20 °C) mixture of methanol and ethanol (1∶1 v/v). Samples were vortex-mixed and then stored at − 20 °C for 15 min. After this, the pellet was removed by centrifuging at 16,000×g for 10 min at 4 °C. Next, the supernatant was filtered through a nylon filter (0.22 μm). The solution obtained was injected into the chromatographic column to undergo quantitation with LCMS analysis.

##### SFPE procedures

*HPTLC plate preparation*.

HPTLC Diol F254 10 × 20 cm plates were cut into 10 × 10 cm sheets using TLC plate cutter (CAMAG). Before chromatogram development the plates were washed in methanol for 1 min. Next, the plates were dried in the air and activated in an oven at 105–110^◦^C for 15 min^31–33,35^.

*Samples application on the HPTLC plate*.

The samples were applied as a small single drops on the chromatographic plate by using the automatic pipette (PZ HTL S.A., Warsaw, Poland). The volume of drop was about 2 µL.

*Equipment for SFPE procedure*.

Thin-layer chromatography experiments were conducted using a prototype device equipped with moving pipette, worked out in the department, driven by 3D machine (Infinum 3D, Lublin, Poland). The device enabled to deliver the solution of the mobile phase to the adsorbent layer with controlled velocity^35^. The results were documented using the TLC Visualizer (CAMAG, Muttenz, Switzerland). Extraction the substances was performed using the CAMAG TLC–MS Interface, connected with Agilent 1260 Infinity Isocratic pump.

*Chromatograms development*.

Single drops (2µL) of sample were spotted on the chromatographic plates as two start lines, located at a distance 37 mm and 63 mm from the chromatographic plate edge. Then, the plate was left for 10 min to dry the spots of sample. The path of the pipette delivering the mobile phase to the adsorbent layer was located in the middle of the chromatographic plate between the starting lines. The solvents used for chromatogram development were selected according to the SFPE procedure rules (see section “SFPE procedure optimization” for details). The distance of the first development with a solution of 0.1% formic acid in methanol was 18–20 mm (4–5 mm behind of samples zones). In the next step, the chromatogram was developed, on distance from 20 to 22 mm, with a solution of 35% toluene in methanol (with 0.1% addition of formic acid). Then the two paths of the pipette movement were located parallel to the starting lines at a distance 16 mm from the middle of the chromatographic plate. The speed, F, of pipette movement was established as 3000 mm/min, the efficiency of the mobile phase delivery to the layer of adsorbent: 6 mL/h. When this procedure step was completed the plate was dried in the air for 5 min. In the next step, the chromatogram was developed at distance from 10 to 12 mm, with the 0.1% formic acid solution in methanol. The two paths of the pipette movement were located parallel to the starting lines at a distance 16 mm from the middle of chromatographic plate. F = 3000 mm/min, the efficiency of the mobile phase solution delivery to the adsorbent layer: 6 mL/h. The last stage brought the substance zones to a common place in the solvent front position^30,31,35^.

*Extraction of the substances from the chromatographic plate*.

After evaporation of solvent from the chromatographic plates the substances were extracted from solvent front position using the TLC–MS Interface (CAMAG, Muttenz, Switzerland) and Agilent 1260 Infinity Isocratic Pump. The extracting solvent was 0.1% formic acid in water. Flow velocity of the solvent was 0.3 mL/min. The obtained samples were subjected to three variants of instrumental analysis: (1) the samples were collected in vials and transferred to HPLC combined with MS, variant denoted as “SFPE-vials-LCMS”; (2) collected in vials and transferred to MS, denoted as “SFPE-vials-MS”; (3) the samples were directly transferred (online) with the TLC-MS Interface to MS, denoted as “SFPE-MS”.

### LC–MS/MS analysis

The Agilent 1290 Infinity LC System (Santa Clara, United States) connected with Agilent 6460 Triple Quadrupole was used for the LC–MS experiments. The chromatography (classical and SFPE-vials-LCMS procedures) was performed with the Agilent Poroshell 120 SB-Aq column (2.1 × 100 mm, 2.7 µm). Mobile phase was consist of: A: 0.1% formic acid in water and B: 0.1% formic acid in ACN. The gradient elution was conducted as follows: 0 min: 99% A, 1% B; 1 min: 99% A, 1% B; 5 min: 50% A, 50% B; 5.1 min: 5% A, 95% B; 8 min: 5% A, 95% B, next 4 min re-equilibration with the starting mobile phase. Mobile phase flow rate: 0.4 mL/min. In case of SFPE-vials-MS and SFPE-MS procedures the extracting mobile phase was comprised of 0.1% formic acid in water, flow rate: 0.4 mL/min.

MS data were acquired in the positive ion modes (multiple-reaction monitoring mode) with electrospray probe voltages of 3500 V. Nebulizer gas setting was 30 psi. The ion source was operated at temperature 250 °C and a drying gas setting 11 L/min.

### Ethics approval and consent to participate

All experiments and methods were performed in accordance with relevant guidelines and regulations. The protocols were approved by the Bioethics Committee at the Medical University of Lublin. Informed consent was received from all participants in the study.

## Conclusion

We have previously shown that the SFPE procedure can be effectively used to determine selected substances in fortified serum samples. The results presented in current paper prove that the SFPE technique of sample preparation can be successfully applied to analysis of real clinical samples. Quantitative results obtained using this procedure do not differ from those obtained using the classic, well-established sample preparation procedure of protein precipitation. Furthermore, the sample prepared by the SFPE procedure is more purified from matrix components in comparison to the samples prepared by the precipitation procedure. Due to that advantage it is possible to skip the column chromatography step, what significantly reduces the time and cost of the analysis (SFPE-vials-MS and SFPE-MS procedures). In our opinion especially the merits of the SFPE-MS procedure are noteworthy. Due to its lower cost and the shortest analysis time as well as the ease of full automation that method can be very competitive of the currently used methods of sample preparation of biological origin.
